# Simples

**Published:** 1878-01

**Authors:** 


### SIMPLES.

There are simple drugs and simple brains, the latter having the majority. "It can do no harm, if it does no good," is a simpleton's speech about a "simple remedy." Let us see if this will bear investigation.

One of the sweetest "seventeens" we know of, in driving a nail on which to hang a canary cage, hit her finger instead of the nail—a thing not unfrequently done. In fact, a multitude, which no man can number, fail to hit the nail on the head, as to the great object of life! In a few moments, she began to practice a piece of piano music, which added to the injury, and in the course of the evening, the finger became painful. A friend advised to poultice it with the white of an egg, which, in [illegible] might be taken into the mouth and passed along, even to advantage, if a man was hungry. She retired, but spent a night of agony; the whole house was in commotion, and the next day we were called on to prescribe for a felon, the safest treatment for which, if early, is to drive a lancet to the bone, and scrape upon it, so as to be sure of having gone deep enough. But to treat the pretty finger of a pretty patient in that way, was not to be thought of, until the last resort. Upon cross-examination, as necessary in physic as in law, all the above facts were ferreted out, when we concluded that there was nothing the matter but fright and the white of an egg. Thus: it had dried over the skin of the finger-end and became as impervious to the exhalation of that heat and moisture which pass out of the system unceasingly, as if it had been hermetically sealed with an encasing of brass. The result was, the heat and fluids accumulated, the parts became dry and hot and hard, and the pain became as unendurable as a pirate's thumb-screw.

MORAL.—"Simples" are only simple when in their proper place; and the familiar quotation, "*It can do no harm* if it does no good," is, in medicine at least, a dangerous untruth.

Reader! Let us give you the most wholesome piece of advice of the season—

*Do nothing remedially without your Doctor's consent.*

